# Inverse association between pre-high-altitude potassium levels and coronary microvascular disease at high altitude: a case-control study

**DOI:** 10.3389/fphar.2025.1659041

**Published:** 2025-08-21

**Authors:** Yu Chen, Yong Ren, Xin Zhang, Wen-Bao Zhou, Wen-Tao Zhang, Juan Lv, Xi-Yang Rao, Rui-Xi Zi, Si-Jie Wang, Li-Sha Wang, Chun-Ping Bao, Li-Xia Yang, Gong-Hao He, Yan-Kun Shi

**Affiliations:** ^1^ Department of Cardiology, 920th Hospital of Joint Logistics Support Force of PLA, Kunming, China; ^2^ Department of Cardiology, Daping Hospital, The Third Military Medical University (Army Medical University), Chongqing, China; ^3^ Department of Clinical Medical College, Dali University, Dali, China; ^4^ Department of Pulmonary and Critical Care Medicine, 920th Hospital of Joint Logistics Support Force of PLA, Kunming, China; ^5^ Department of Health Medicine, 920th Hospital of Joint Logistics Support Force of PLA, Kunming, China; ^6^ Department of Clinical Pharmacy, 920th Hospital of Joint Logistics Support Force of PLA, Kunming, China

**Keywords:** altitude exposure, coronary microvascular disease, risk factors, epidemiology, case-control study

## Abstract

**Background and Objectives:**

High-altitude hypoxia is known to impair cardiac microvascular function, a pathophysiological state recognized as coronary microvascular disease (CMVD). This study aimed to investigate the independent association between serum potassium levels and the risk of developing CMVD, after controlling for traditional cardiovascular risk factors such as hypertension, dyslipidemia, and smoking.

**Methods:**

This case-control study enrolled 1,175 trainees with long-term exposure to high altitude (≥3,000 m), comprising 235 patients with CMVD (cases) and 940 healthy controls. We employed multivariable logistic regression analysis to systematically evaluate the associations of traditional risk factors and serum potassium levels with the risk of CMVD.

**Results:**

A key finding from our analysis was a significant inverse association between serum potassium levels and the odds of CMVD (OR = 0.26; 95% CI, 0.14–0.47). Specifically, higher potassium concentrations were correlated with a substantially lower disease risk. This inverse association was more prominent in individuals with a moderate body mass index and in smokers.

**Conclusion:**

This is the first study to demonstrate that a higher serum potassium level is independently associated with lower odds of CMVD in populations exposed to high altitudes. This finding provides a new direction for developing targeted health screening and preventive strategies in high-altitude regions, holding significant potential for protecting specific at-risk groups such as individuals with a particular BMI, and smokers.

## 1 Introduction

Coronary microvascular disease (CMVD) has emerged as a significant contributor to cardiovascular morbidity, particularly in angina patients without obstructive Coronary Artery Disease (CAD) (Crea et al., 2022). This condition reflects coronary microvascular dysfunction, leading to myocardial ischemia and reduced quality of life. The pathophysiology of CMVD is complex, involving various mechanisms such as endothelial dysfunction, inflammation, and altered vasomotor responses that impact coronary blood flow regulation (He et al., 2021). Despite increasing recognition of its clinical importance, effective treatments for CMVD remain limited, underscoring the need for a deeper understanding of its underlying causes and potential therapeutic targets (Al-Mohaissen, 2023).

Understanding CMVD risk factors and their interactions is crucial for developing effective prevention strategies. Previous studies have identified various demographic and physiological risk factors, with certain populations showing increased susceptibility (Gurzau et al., 2021). The diagnosis of CMVD is often challenging due to the absence of significant lesions on angiography, necessitating the use of functional assessments to evaluate coronary microvascular reserve and endothelial function (Vancheri et al., 2020). Recent studies using echocardiography or positron emission tomography (PET) have improved CMVD diagnosis accuracy (Al-Mohaissen, 2023).

Despite this progress, the role of specific environmental factors, such as high-altitude exposure, on the pathogenesis of CMVD has not been extensively studied. High-altitude environments present unique physiological challenges, including hypoxia and reduced barometric pressure, which may further impair coronary microvascular function and increase the risk of cardiovascular events (Mallet et al., 2021). Understanding how these environmental stressors impact microvascular health is crucial, particularly for populations residing in or frequently exposed to high-altitude settings.

Potassium homeostasis is fundamental for maintaining cellular membrane potential, vascular tone, and overall myocardial function, and imbalances have been linked to adverse cardiovascular outcomes. However, the specific relationship between serum potassium levels and the risk of developing CMVD, particularly under the compounding physiological stress of high-altitude exposure, remains a critical knowledge gap. Therefore, this study aimed to investigate the association between pre-exposure serum potassium levels and the risk of CMVD in a large cohort of individuals undergoing high-altitude training. By exploring the interplay between this key electrolyte and microvascular function in a hypoxic environment, our findings may help identify a novel, modifiable risk factor and contribute to improved preventive strategies for CMVD in vulnerable populations.

## 2 Methods

### 2.1 Study design, setting, and population

For this case-control study, we recruited 4,000 subjects (3,800 males, 200 females; aged 18–54 years) between January and June 2022, all of whom were scheduled for deployment to high-altitude regions (3,000–3,500 m).

### 2.2 Participants

A rigorous, multi-stage screening process was applied to the initial cohort. To minimize the influence of pre-existing conditions that could act as significant confounders for cardiovascular function, especially under hypoxic stress, we established strict exclusion criteria based on baseline assessments.

First, we excluded individuals with a history of significant respiratory diseases that could impair oxygenation and induce systemic inflammation, such as pneumonia, asthma, or Chronic Obstructive Pulmonary Disease (COPD) (Bartsch and Gibbs, 2007). Second, we excluded participants with known major cardiovascular diseases or risk factors that are independently associated with microvascular dysfunction. This included a prior diagnosis of systemic hypertension, known coronary artery disease, or significant valvular heart disease (Chen et al., 2023).

Furthermore, all participants underwent baseline electrocardiogram (ECG). Individuals presenting with abnormalities (e.g., ST-T changes, Q waves, significant arrhythmias) or evidence of myocardial hypertrophy were excluded. Participants with ambiguous ECG findings were further evaluated with echocardiography to rule out underlying structural heart disease or pulmonary hypertension. Finally, individuals who did not complete the full high-altitude deployment and those with a documented history of acute mountain sickness, severe injury, or COVID-19 within the preceding 6 months were also excluded. Additionally, individuals who did not ultimately participate in the scheduled high-altitude training within 3 months for various administrative or personal reasons were excluded This comprehensive screening process yielded an eligible group of 2,855 participants.

**FIGURE 1:**
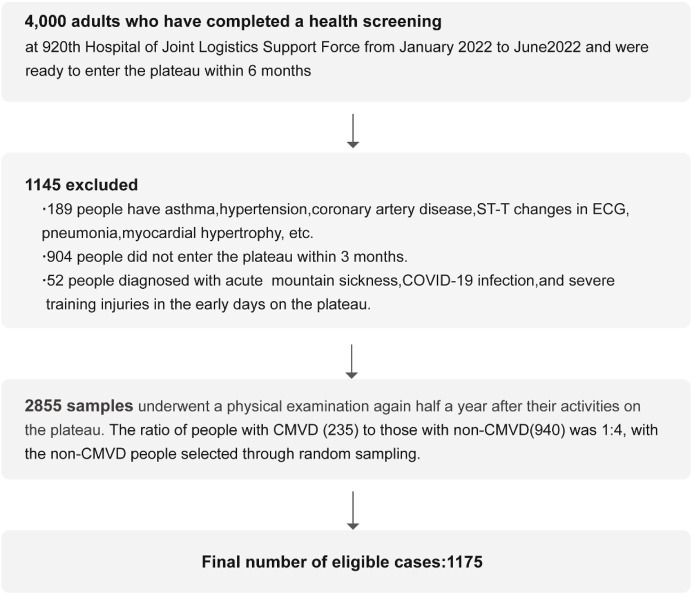
Flowchart of participants selection.

From this eligible group, we identified all 235 incident cases of CMVD, who constituted our ‘case’ group. To assemble a comparable ‘control’ group, we then performed a random sampling procedure on the remaining 2,620 participants who were free of CMVD. From this pool, we randomly selected 940 individuals, establishing a 1:4 case-to-control ratio. It is crucial to clarify that this was a random selection process, and no individual matching based on age, sex, or other parameters was performed. This procedure yielded the final analytical sample of 1,175 participants (Figure 1).

### 2.3 Data collection and procedures

Six months prior to the high-altitude exposure, all participants underwent a systematic baseline health assessment, which included X-ray, electrocardiogram (ECG), ultrasound, and blood tests. Baseline data were collected via an electronic questionnaire, which gathered information on: smoking status and history; recent physical performance scores (3-km run, sit-ups, and serpentine run); clinical symptoms, including chest discomfort or pain; sociodemographic details (residential altitude, education level, marital status); and autonomic symptom burden, as measured by the Scale for Outcomes in Parkinson’s Disease–Autonomic (SCOPA-AUT). To ensure accuracy, the corresponding residential altitude was determined by our research team by cross-referencing this location data with official topographical databases.

A follow-up evaluation was performed at the end of the 6-month deployment at high altitude (December 2022–January 2023). This evaluation comprised repeated clinical assessments (X-ray, ECG), measurement of vital signs (heart rate, blood pressure, and oxygen saturation), hematological analyses, and a follow-up questionnaire.

The SCOPA-AUT (Visser et al., 2004) is a validated, patient-reported instrument designed to quantify the severity of autonomic dysfunction (see Supplementary Material). It assesses a range of non-motor symptoms across key domains such as gastrointestinal, urinary, and cardiovascular regulation. On this scale, higher scores directly correspond to a greater burden of autonomic impairment. All hematological tests were conducted at the medical examination center of the 920th Hospital of Joint Logistics Support Force.

### 2.4 Coronary microvascular disease (CMVD)

All 2,855 subjects first underwent a standard 12-lead electrocardiogram (ECG) to screen for individuals exhibiting signs of myocardial ischemia, such as ST-segment depression or T-wave inversion. Subsequently, these individuals with ischemic ECG findings were further assessed using transthoracic Doppler echocardiography to measure their coronary flow reserve (CFR). A definitive diagnosis of CMVD was made based on the CFR results.

#### 2.4.1 Evaluation of ECG

Participants avoided exercise for 48 h before ECG testing to minimize ST-T changes from physical activity. Myocardial ischemia was diagnosed by ECG showing: 1. Horizontal/downsloping ST depression >0.5 mm at J-point in ≥2 consecutive leads; 2. T wave inversion ≥1 mm in ≥2 continuous leads with R/S > 1 (Brady et al., 2020). Additionally, participants exhibiting abnormal ST-T segments in the electrocardiogram underwent portable echocardiography examinations to exclude ST-T abnormalities attributable to cardiomyopathy or pulmonary hypertension.

#### 2.4.2 Coronary flow reserve (CFR) assessment

Transthoracic Doppler Echocardiography (TDE) assessed CFR in patients with ST-T changes. The mid-distal left anterior descending (LAD) was visualized using a 5–7 MHz probe (Schroder and Prescott, 2021). Adenosine (0.14 mg/kg/min IV) induced hyperemia. Diastolic flow velocities were measured by tissue Doppler, analyzed offline (Carbone et al., 2021). CFR was calculated as hyperemic/baseline flow velocity ratio. A reduction in CFR usually indicates coronary microcirculatory dysfunction, and impaired coronary flow reserve is defined as a CFR ≤2.3 (Gan et al., 2017).

### 2.5 Covariables

We constructed a multivariable logistic regression model to identify factors associated with CMVD, comparing health data collected before and after high-altitude exposure. The selection of covariates was informed by the Chinese expert consensus on the diagnosis and treatment of coronary microvascular diseases (2023 Edition) (Chen et al., 2023), which identifies them as significant risk factors. The final model included the following variables: 1) demographic and clinical parameters: age (Zornitzki et al., 2024), sex (Mygind et al., 2016), body mass index (BMI) (Olsen et al., 2015), and systolic blood pressure (SBP) (Yong et al., 2020); 2) a lipid marker: pre-exposure low-density lipoprotein cholesterol (LDL-C) (Del Buono et al., 2021); 3) a lifestyle factor: smoking status (Rooks et al., 2011) (‘Never’ (never smoked), ‘Quitted’ (stopped smoking for at least 6 months prior to baseline), and ‘Smoking’ (current smokers or those who quit less than 6 months ago)); and 4) autonomic function status (Cattaneo et al., 2022): the post-exposure score on the SCOPA-AUT, a validated tool for quantifying autonomic dysfunction.

### 2.6 Bias

To ensure methodological rigor, random sampling was employed to mitigate selection bias, while the use of standardized scales minimized information bias. To address the issue of classification bias, we implemented a dual-validation system for evaluating Electrocardiogram (ECG) outcomes. Senior cardiologists (>8 years’ experience) supervised the process. TDE assessed CFR in patients with ST-T changes. Currently, invasive coronary angiography remains the gold standard for CMVD diagnosis, measuring CFR and IMR. However, our study could not employ TDE for universal screening nor use invasive coronary angiography for definitive diagnosis. Consequently, there remains a certain discrepancy between our diagnostic outcomes and this gold standard.

### 2.7 Study size

Our study utilized an available cohort of personnel, and therefore a formal a priori sample size calculation was not performed to determine the initial number of participants. In multivariable analyses, each variable’s effect depends on other covariates. The most stringent empirical rule suggests a sample size that is 20 times the number of variables (Norman et al., 2012). In our case, we included 8 variables in the final model (Table 2). The robustness of the final multivariable logistic regression model was rigorously assessed. With 235 outcome events and 7 predictor variables incorporated into the model, the resulting Events Per Variable (EPV) was 33.6. This figure far exceeds the conventional threshold of ≥10 EPV (Norman et al., 2012), a widely accepted criterion for preventing model overfitting. Therefore, the high EPV confirms the stability of our model and minimizes the risk of spurious findings, lending strong support to the reliability of our results.

### 2.8 Statistical methods

Continuous variables were presented as mean (SD) for normal distributions and median (interquartile range) for non-normal distributions. Statistical comparisons were performed using Student's t-test for normally distributed data, Mann-Whitney U test for non-normal distributions, and chi-square test for categorical variables. Statistical significance was defined as p < 0.05.

To assess the independent association between pre-high-altitude serum potassium levels and the risk of CMVD, we constructed a multivariable logistic regression model. Based on established risk factors identified in the literature, we pre-selected a comprehensive set of potential confounders to be included in the model. These variables were: age, sex, Body Mass Index (BMI), SBP, pre-exposure low-density lipoprotein cholesterol (LDL-C), smoking status, and the SCOPA-AUT score (post-high altitude). In the multivariable logistic regression analysis, the variables for K (pre-high altitude), age, BMI, SBP, LDL-C, and SCOPA-AUT score (post-high altitude) were all entered into the model as continuous variables. The resulting odds ratios (ORs) therefore represent the change in odds for a one-unit increase in each respective variable. All these variables were included in the final model regardless of their statistical significance in the univariate analysis.

The preliminary model was subsequently assessed for multicollinearity. All predictor variables exhibited a Variance Inflation Factor (VIF) well below the established threshold of 5 (Fox and Monette, 1992), confirming the absence of significant collinearity issues. Odds ratios (ORs) and their 95% confidence intervals (CIs) were calculated using multivariable unconditional logistic regression (Walter, 1975). This method is appropriate for our study design, as the cases and controls represent two independent groups assembled through random sampling, rather than individually matched pairs or strata. Statistical analyses were performed using R version 4.2.0.

## 3 Results

### 3.1 Participants

The detailed process of participant selection and exclusion is described in the Methods section and visually summarized in the flowchart in Figure 1. After the screening process, a total of 2,855 participants (2,810 males, 45 females) were included in the follow-up. In the medical examinations conducted after 6 months of deployment at the high-altitude area, among these individuals, 235 were subsequently diagnosed with CMVD and designated as cases. From the remaining 2,620 individuals without CMVD, 940 were randomly selected as controls. This resulted in a final analytical sample of 1,175 individuals for this study.

### 3.2 Descriptive data

Baseline demographic and clinical characteristics are shown in Table 1. The group consisted of 1,175 participants, with 235 individuals presenting with CMVD and 940 without. The median age was comparable between groups: 24.0 years [22.0; 29.0] for those with CMVD and 24.0 years [22.0; 27.0] for those without (p = 0.014). A significant difference was observed in body mass index (BMI), with the CMVD group having a slightly higher BMI of 22.6 [20.9; 24.4] compared to 22.1 [20.8; 23.5] in the control group (p = 0.019).

**TABLE 1:** Baseline characteristics for cases and controls of CMVD.

	Cases	Controls	p-value
	N = 235	N = 940	
Age (years)	24.0 [22.0; 29.0]	24.0 [22.0; 27.0]	0.014
BMI (kg/m^2^)	22.6 [20.9; 24.4]	22.1 [20.8; 23.5]	0.019
Sex			0.016
Female	8 (3.40%)	10 (1.06%)	
Male	227 (96.6%)	930 (98.9%)	
SBP (mmHg)	119 [110; 125]	117 [110; 120]	0.001
OS (%)	93.0 [92.0; 95.5]	93.0 [92.0; 95.0]	0.035
K (pre-high-altitude) (mmol/L)	3.87 [3.73; 3.96]	3.94 [3.74; 4.12]	<0.001
K (post-high-altitude) (mmol/L)	3.70 [3.34; 4.12]	3.83 [3.46; 4.21]	0.004
LDL-C (pre-high altitude) (mmol/L)	2.90 (0.43)	2.91 (0.49)	0.642
Smoking status			0.360
Never	106 (45.1%)	444 (47.2%)	
Quitted	14 (5.96%)	76 (8.09%)	
Smoking	115 (48.9%)	420 (44.7%)	
SCOPAAUT SCORE (post-high altitude)	5.00 [3.00; 9.00]	6.00 [3.00; 10.0]	0.560

Continuous data conforming to a normal distribution were reported as mean (SD) (for normally distributed variables: LDL-C), those not conforming as median [quartiles] (for skewed continuous variables: Age, BMI, SBP, OS, K (pre-high altitude), K (post-high altitude), LDL-C (pre-high altitude), SCOPAAUT SCORE), and number (percentage) (for categorical variables: Sex, Smoking status). Statistical significance was determined using t-test, Rank-sum test or χ^2^ test, with p-values calculated.

The “Cases” column represents participants diagnosed with CMVD (cases, N = 235), and the ‘Control’ column represents participants without CMVD (controls, N = 940).

BMI: body mass index; OS: oxygen saturation. High Altitude: an altitude≥2500 m. LDL: Low-Density Lipoprotein Cholesterol; SBP: systolic blood pressure. SCOPA-AUT: the Scale for Outcomes in Parkinson′s Disease for Autonomic Symptoms. Smoking Status: ‘Never’ (never smoked), ‘Quitted’ (stopped smoking for at least 6 months prior to baseline), and ‘Smoking’ (current smokers or those who quit less than 6 months ago).

Sex distribution showed a significant association, where males constituted 96.6% of the CMVD group compared to 98.9% in the non-CMVD group (p = 0.016). Systolic blood pressure (SBP) was slightly elevated in the CMVD group (119 [110; 125] mmHg vs. 117 [110; 120] mmHg, p = 0.001). Serum potassium (K) levels Pre-High-Altitude and After-High-Altitude also showed significant differences, with the CMVD group having lower median potassium levels in Pre-High-Altitude (3.87 [3.73; 3.96] vs. 3.94 [3.74; 4.12], p < 0.001) (Supplementary Figure S1) and After-High-Altitude (3.70 [3.34; 4.12] vs. 3.83 [3.46; 4.21], p = 0.004).

Smoking status did not show a statistically significant difference between groups (p = 0.360), though there was a trend of higher smoking rates in the CMVD group (48.9%) compared to the non-CMVD group (44.7%).

### 3.3 Outcome data

Univariate and multivariable logistic regression analyses were performed to identify risk factors for CMVD at high altitude.

Univariate Analysis: Key variables that emerged as independent risk factors included age, BMI, Sex, SBP, K (pre-high altitude). Specific odds ratios (OR) and 95% confidence intervals (CI) for these factors are detailed in Table 2.

**TABLE 2:** Univariate and multivariable binary logistic regression analyses for the relationship between CMVD following high-altitude exposure and clinical characteristics.

Variables	Univariate		Multivariable	
	OR (95% CI)	p value	OR (95% CI)	p-value
K (pre-high altitude)	0.25 (0.14–0.45)	p <0 .001	0.26 (0.14–0.47)	p <0.001
Age	1.05 (1.02–1.08)	p = 0.002	1.03 (1.00–1.06)	p = 0.068
BMI	1.09 (1.02–1.16)	p = 0.009	1.06 (1.00–1.14)	p = 0.068
SBP	1.02 (1.01–1.03)	p = 0.002	1.02 (1.00–1.03)	p = 0.012
Sex				
Male				
Female	3.28 (1.28–8.40)	p = 0.013	3.19 (1.19–8.50)	p = 0.021
LDL-C (pre-high altitude)	0.94 (0.69–1.26)	p = 0.665		
Smoking status				
Never				
Quitted	0.77 (0.42–1.42)	p = 0.403		
Smoking	1.15 (0.85–1.54)	p = 0.364		
SCOPAAUT SCORE (post-high altitude)	0.99 (0.97–1.02)	p = 0.657		

OR: odds ratio. CI: confidence interval; SCOPA-AUT: the Scale for Outcomes in Parkinson′s Disease for Autonomic Symptoms.

**FIGURE 2:**
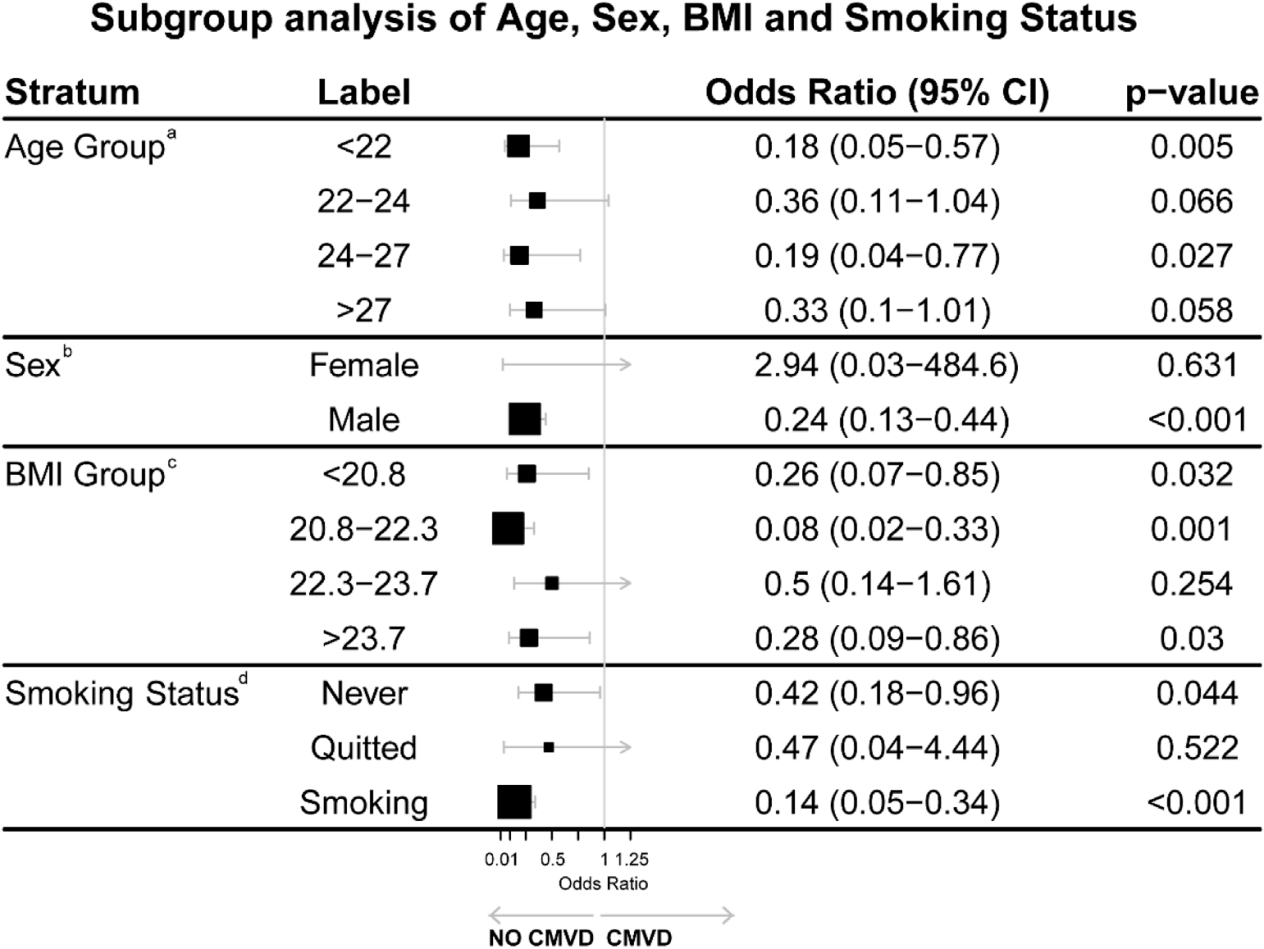
Subgroup analysis of Age, Sex, BMI, and Smoking Status. Both age and BMI were divided into quartiles for detailed analysis. ^a^OR of Age Group adjusted for BMI, SBP, LDL-C pre-high altitude, smoking status and SCOPAAUT SCORE post-high altitude. ^b^OR of Sex adjusted for no covariant. Due to the very small sample size of female participants, this result is statistically unreliable and likely a spurious finding resulting from the low number of events in this subgroup. ^c^OR of BMI Group adjusted for Age, SBP, LDL-C pre-high altitude, Smoking status and SCOPAAUT SCORE post-high altitude. ^d^OR of Smoking status adjusted for Age, SBP, LDL-C pre-high altitude, and SCOPAAUT SCORE post-high altitude.

Multivariable Analysis: Among the variables analyzed, higher pre-high-altitude potassium (K) levels were significantly associated with lower odds of CMVD (OR = 0.26, 95% CI: 0.14–0.47). In contrast, female sex emerged as a significant risk factor with an OR of 3.19 (95% CI: 1.19–8.50). These findings are illustrated in Table 2 and Figure 2.

### 3.4 Subgroup analysis

A subgroup analysis (Figure 2) was conducted to explore the associations between various covariates and the likelihood of developing cardiometabolic vascular disease (CMVD). Both age and BMI were divided into quartiles for detailed analysis.

Age: Compared with participants aged >27 years, those aged <22 years (OR: 0.18, 95% CI: 0.05–0.57, P = 0.005) and 24–27 years (OR: 0.19, 95% CI: 0.04–0.77, P = 0.027) had lower risks of CMVD. The risk reduction in the 22–24 years group was not significant (OR: 0.36, 95% CI: 0.11–1.04, P = 0.066). Individuals over 27 years did not show a statistically significant difference (p = 0.058).

Sex: Males exhibited a significantly lower risk of developing CMVD compared to females (OR: 0.24, 95% CI: 0.13–0.44, p < 0.001), though the result for females showed wide confidence intervals (OR: 2.94, 95% CI: 0.03–484.6, p = 0.631), likely due to a smaller sample size in this subgroup.

BMI: Compared with participants with BMI >23.7, those with BMI 20.8–22.3 (OR: 0.08, 95% CI: 0.02–0.33, P = 0.001) and <20.8 (OR: 0.26, 95% CI: 0.07–0.85, P = 0.032) showed lower risks of CMVD. No significant differences were found in the BMI 22.3–23.7 group (P = 0.254).

Smoking Status: Compared with former smokers, current smokers (OR: 0.14, 95% CI: 0.05–0.34, P < 0.001) and non-smokers (OR: 0.42, 95% CI: 0.18–0.96, P = 0.044) had lower risks of CMVD.

## 4 Discussion

Coronary microvascular disease (CMVD) is characterized by coronary microvasculature dysfunction, causing myocardial ischemia in patients without obstructive coronary artery disease (Szolc et al., 2024). Patients typically present with microvascular angina - chest pain due to impaired microvascular blood flow despite normal epicardial coronary arteries (Waheed et al., 2020). CMVD predominantly affects women and patients with diabetes or hypertension, increasing their risk of adverse cardiovascular events (Wu et al., 2020). Its pathophysiology involves systemic inflammation and endothelial dysfunction (Ferrannini et al., 2021). In this context, the present study investigates the association of pre-high-altitude serum potassium levels on CMVD risk in individuals undergoing high-altitude training. Hypokalemia affects cardiac function and is associated with cardiovascular diseases, including CMVD (Crea et al., 2022). Utilizing a case-control study design with 1,175 participants, this research aims to elucidate the relationship between pre-training serum potassium levels in assessing CMVD risk, particularly in the context of high-altitude exposure. The findings from this study may offer new insights into the mechanisms of CMVD under high-altitude conditions and contribute to the development of personalized preventive strategies for high-risk populations (Kumar et al., 2021; Omuro et al., 2024).

This study explored the association between serum potassium levels and the risk of coronary microvascular disease (CMVD) in a high-altitude setting, focusing on CMVD as a key driver of myocardial ischemia in individuals with patent coronary arteries. Our principal finding is that higher pre-exposure serum potassium levels are significantly associated with a lower risk of CMVD. This association may be underpinned by several plausible biological mechanisms. First, potassium may exert a direct vasodilatory effect. In hypoxic conditions, the depletion of cellular ATP activates ATP-sensitive potassium (KATP) channels (Salomonsson et al., 2017). This leads to membrane hyperpolarization, which subsequently induces the relaxation of vascular smooth muscle, thereby directly enhancing myocardial perfusion. Second, potassium likely attenuates endothelial dysfunction. By stabilizing the cellular electrophysiological milieu, potassium can mitigate hypoxia-induced oxidative stress and inflammatory responses, thus safeguarding the vascular endothelium from injury. Third, it appears to act in synergy with the nitric oxide (NO) pathway (Fondjo et al., 2023). Potassium-mediated hyperpolarization enhances both the synthesis and release of NO, creating a more potent vasodilatory effect in concert with the NO pathway. The clinical implications of these findings are profound. Identifying serum potassium as a modifiable protective factor could inform the development of targeted screening protocols for populations undertaking missions at high altitudes, such as athletes and military personnel. Given the heightened cardiovascular risk in these specialized groups, integrating potassium surveillance into routine pre-deployment evaluations could facilitate early identification and intervention for at-risk individuals, potentially mitigating adverse cardiac events during high-altitude exposure.

In our BMI-stratified analysis, the inverse association between potassium and CMVD was strongest in the moderate BMI group (20.8–22.3), with an odds ratio of 0.08 (95% CI: 0.02–0.33, p = 0.001). This inverse association was relatively weaker in both lower BMI (<20.8, OR: 0.26, 95% CI: 0.07–0.85, p = 0.032) and higher BMI groups (>23.7, OR: 0.28, 95% CI: 0.09–0.86, p = 0.03). The stronger inverse association between potassium and CMVD in the moderate BMI group may be attributable to an optimal body composition. This could reflect a favorable balance between lean muscle mass and adipose tissue, which is crucial for potassium homeostasis and microvascular function. Skeletal muscle, serving as the primary potassium reservoir, maintains stable potassium levels during high-altitude exposure, while better insulin sensitivity in this BMI range facilitates cellular potassium uptake and endothelial function. The weaker association in lower BMI groups might stem from reduced muscle mass and thus a smaller potassium storage capacity. Meanwhile, the altered adipokine profiles and increased inflammatory markers common in higher BMI groups could counteract or obscure the statistical link between potassium and CMVD risk.

Both BMI extremes show altered sympathetic nervous system activity (Bagi et al., 2014), potentially affecting potassium handling and microvascular tone under high-altitude stress. Our findings suggest that the inverse association between serum potassium and CMVD is most pronounced in individuals with a BMI between 20.8 and 22.3. This highlights the potential importance of weight management for individuals preparing for high-altitude exposure, as maintaining a BMI in this range may create a more favorable physiological environment for microvascular health.

Our analysis stratified by smoking status indicated that the inverse association between potassium and CMVD was substantially stronger in current smokers (OR: 0.14, 95% CI: 0.05–0.38, p < 0.001) compared to never-smokers (OR: 0.42, 95% CI: 0.19–0.91, p = 0.03).

This seemingly counterintuitive finding might be explained by several mechanisms. Chronic smoking alters ion channel expression and function in vascular tissue, potentially leading to upregulation of certain potassium channels as a compensatory response (Le et al., 2021). Additionally, smokers’ heightened sympathetic activity and enhanced stress responses might amplify the beneficial effects of adequate potassium levels during high-altitude adaptation. The weaker inverse association observed in never-smokers likely reflects their better baseline microvascular function and more robust physiological reserves, making the incremental benefit of higher potassium levels less pronounced. The interaction between smoking and potassium homeostasis becomes particularly relevant in high-altitude settings, where smoking affects both pulmonary function and tissue oxygenation. The stronger inverse association between potassium and CMVD in smokers may reflect a heightened biological need to counteract smoking-induced microvascular dysfunction. This underlying vulnerability is likely exacerbated by the physiological stress of high altitude, thus making the role of potassium in maintaining vascular health more prominent. However, this finding must not be misconstrued as a justification for smoking. The well-established and overwhelming adverse health consequences of smoking far outweigh the observation of a modified statistical association between potassium and CMVD in this specific high-risk group.

Despite the significant findings and strengths of this study, several limitations must be acknowledged. First, the primarily observational and case-control study design restricts our ability to draw causal inferences between pre-training serum potassium levels and CMVD risk. Second, our model identified female sex as a variable with a significant odds ratio. However, this must be considered a major limitation of our study. The cohort was predominantly male, and the number of female participants was far too small to support any conclusions about sex-based differences in CMVD risk. While some larger epidemiological studies with more balanced populations have suggested that female sex is an independent risk factor for CMVD (Mileva et al., 2022), our study lacks the statistical power to contribute to that discussion. This highlights the need for future research in more diverse and representative cohorts. Third, the study specifically targeted individuals engaged in high-altitude training, which may not fully represent the general population at risk for CMVD. The absence of longitudinal data further restricts our ability to assess how serum potassium fluctuations correlate with long-term outcomes in CMVD patients, making it challenging to evaluate the temporal relationship between potassium levels and disease progression. Moreover, the variability in individual physiological responses to high-altitude environments—such as genetic predisposition, pre-existing health conditions, and lifestyle choices—complicates the interpretation of our results.

Future research should address these limitations by including more diverse populations and employing longitudinal study designs to better elucidate the relationship between serum potassium levels and CMVD risk over time. Integrating physiological assessments of potassium metabolism alongside serum levels could yield deeper insights into the pathophysiology of CMVD. Comprehensive evaluations of microvascular function and its correlates would provide a more nuanced understanding of the mechanisms underlying CMVD development. Thus, while this study lays a foundation for future inquiries, it also underscores the complex interplay of metabolic and environmental factors in cardiovascular health, particularly within the context of high-altitude exposure.

## Data Availability

The raw data supporting the conclusions of this article will be made available by the authors, without undue reservation.

## Abbreviations

BMI, body mass index; CMVD, Coronary microvascular disease; K, Potassium; LDL-C, Low-Density Lipoprotein Cholesterol; OS, oxygen saturation; SBP, systolic blood pressure; SCOPA-AUT, the Scale for Outcomes in Parkinson′s Disease for Autonomic Symptoms.
