# Supplementary material for: Inverse association between pre-high-altitude potassium levels and coronary microvascular disease at high altitude: a case-control study
Source: Front Pharmacol. 2025 Aug 21;16:1659041. doi: 10.3389/fphar.2025.1659041 (PMC12408258; doi:10.3389/fphar.2025.1659041)
Supplement: Supplementary file 1 [file Supplementaryfile1.docx]

**Table S1. Characteristics of CMVD cases and their controls**

|  | **CMVD** | |  |
| --- | --- | --- | --- |
|  | ***Cases*** | ***Controls*** | **P Value** |
|  | ***N=235*** | ***N=940*** |  |
| Age(year) | 24.0 [22.0;29.0] | 23.0 [22.0;26.0] | <0.001 |
| Sex: |  |  | 0.050 |
| Female | 8 (3.40%) | 13 (1.38%) |  |
| Male | 227 (96.6%) | 927 (98.6%) |  |
| BMI(kg/cm^2^) | 22.6 [20.9;24.4] | 22.2 [21.0;23.7] | 0.088 |
| SBP(mmHg) | 119 [110;125] | 116 [110;120] | 0.002 |
| DBP(mmHg) | 80.0 [75.0;86.0] | 80.0 [73.0;84.0] | 0.030 |
| MAP(mmHg) | 93.0 [87.0;98.5] | 92.0 [86.0;95.0] | 0.003 |
| HR（min^−1^） | 80.0 [72.0;87.5] | 80.0 [70.0;88.0] | 0.569 |
| OS(%) | 93.0 [92.0;95.5] | 93.0 [92.0;95.0] | 0.041 |
| Highland acclimatization training* |  |  | 0.644 |
| No | 16 (6.81%) | 54 (5.74%) |  |
| Yes | 219 (93.2%) | 886 (94.3%) |  |
| Altitude of hometown(m) | 161 [37.0;364] | 157 [40.0;391] | 0.510 |
| Smoking status |  |  | 0.110 |
| Never | 106 (45.1%) | 452 (48.1%) |  |
| Quitted | 14 (5.96%) | 87 (9.26%) |  |
| Smoking | 115 (48.9%) | 401 (42.7%) |  |
| Smoking duration |  |  | 0.011 |
| 0 year | 120 (51.1%) | 539 (57.3%) |  |
| <1 year | 8 (3.40%) | 46 (4.89%) |  |
| 1-9 years | 82 (34.9%) | 299 (31.8%) |  |
| 10-19 years | 23 (9.79%) | 56 (5.96%) |  |
| 20-39 years | 2 (0.85%) | 0 (0.00%) |  |
| Scores of FTND | 0.00 [0.00;1.00] | 0.00 [0.00;0.00] | 0.006 |
| 3km test in Non-High Altitude (Score)^#^ | 82.0 [55.0;93.0] | 77.0 [65.0;91.0] | 0.275 |
| Sit-ups in Non-High Altitude (Score)^#^ | 95.0 [87.5;101] | 93.0 [85.0;101] | 0.496 |
| Serpentine Run in Non-High Altitude (Score)^#^ | 89.0 [77.0;96.0] | 90.0 [75.0;96.0] | 0.858 |
| Global PSQI Score in Non-High Altitude | 4.00 [2.00;5.00] | 3.00 [1.00;5.00] | 0.532 |
| SCOPA-AUT SCORE in Non-High Altitude | 4.00 [2.00;8.00] | 4.00 [2.00;8.00] | 0.346 |
| A/G | 1.70 [1.50;1.80] | 1.90 [1.60;2.10] | <0.001 |
| ALB(g/L) | 46.5 [45.1;48.1] | 47.3 [45.1;48.8] | 0.017 |
| ALP(U/L) | 62.0 [50.0;67.5] | 59.0 [51.0;73.0] | 0.020 |
| ALT(U/L) | 19.0 [15.0;30.0] | 18.0 [15.0;28.0] | 0.582 |
| AST(U/L) | 20.0 [18.0;25.0] | 22.0 [19.0;30.0] | <0.001 |
| Baso(*10^9^/L) | 0.03 [0.02;0.04] | 0.02 [0.02;0.03] | 0.011 |
| Baso.Per(%) | 0.50 [0.30;0.60] | 0.40 [0.30;0.60] | 0.002 |
| Ca(mmol/L) | 2.42 [2.32;2.47] | 2.37 [2.31;2.41] | <0.001 |
| CHE(U/L) | 8771 [7474;10545] | 8705 [7370;9258] | 0.134 |
| Cl(mmol/L) | 102 [101;103] | 101 [100;103] | <0.001 |
| CO_2_CP(mmol/L) | 27.6 [26.4;28.5] | 27.1 [25.0;28.2] | 0.004 |
| Crea(μmol/L) | 84.0 [79.0;94.5] | 88.0 [81.0;97.0] | <0.001 |
| CysC(mg/L) | 0.90 [0.81;0.95] | 0.88 [0.82;0.92] | 0.895 |
| DBIL(μmol/L) | 5.20 [4.00;6.45] | 4.20 [3.60;6.40] | 0.002 |
| Eo(*10^9^/L) | 0.15 [0.07;0.37] | 0.15 [0.09;0.20] | 0.039 |
| Eos.Per(%) | 2.50 [1.20;5.00] | 2.20 [1.60;3.00] | 0.001 |
| G(g/L) | 27.8 [25.6;28.5] | 25.6 [23.5;27.3] | <0.001 |
| GGT(U/L) | 18.0 [14.0;30.0] | 16.0 [13.0;23.0] | 0.029 |
| GLU(mmol/L) | 4.33 [4.10;4.59] | 4.01 [3.53;4.47] | <0.001 |
| HCT(L/L) | 45.7 [44.3;46.9] | 45.4 [43.7;48.0] | 0.263 |
| HGB(g/L) | 150 [148;158] | 153 [146;162] | 0.528 |
| K(mmol/L) | 3.87 [3.73;3.96] | 3.92 [3.74;4.12] | <0.001 |
| LCR.Per(%) | 26.5 [21.4;33.4] | 25.6 [20.6;27.2] | <0.001 |
| LyM(*10^9^/L) | 2.10 [1.75;2.44] | 2.42 [2.10;2.62] | <0.001 |
| Lymph.Per(%) | 34.0 [30.9;39.3] | 36.9 [32.5;39.2] | 0.002 |
| MCH(pg) | 29.9 [29.0;31.0] | 29.9 [29.4;31.5] | <0.001 |
| MCHC(g/L) | 332 [328;340] | 341 [331;344] | <0.001 |
| MCV(fL) | 90.3 [86.5;92.0] | 88.9 [87.5;91.6] | 0.757 |
| Mono(*10^9^/L) | 0.42 [0.35;0.48] | 0.42 [0.39;0.52] | 0.004 |
| Mono.Per(%) | 6.60 [5.50;7.80] | 6.70 [5.40;7.60] | 0.605 |
| MPV(fL) | 10.2 [9.50;11.1] | 10.0 [9.20;10.2] | <0.001 |
| Na(mmol/L) | 144 [143;144] | 144 [143;146] | 0.243 |
| Neu(*10^9^/L) | 3.16 [2.78;4.15] | 3.72 [3.08;4.16] | <0.001 |
| Neu.Per(%) | 53.5 [50.5;57.2] | 55.3 [49.8;58.0] | 0.881 |
| P(mmol/L) | 1.09 [1.05;1.14] | 1.19 [1.07;1.27] | <0.001 |
| PA(mg/L) | 313 [293;341] | 312 [290;334] | 0.137 |
| PCT | 0.22 [0.18;0.24] | 0.22 [0.20;0.25] | 0.014 |
| PDW(%) | 15.9 [13.1;16.3] | 16.0 [11.9;16.1] | 0.073 |
| PLT(*10^9^/L) | 220 [171;242] | 228 [210;248] | <0.001 |
| RBC(*10^9^/L) | 5.10 [4.86;5.39] | 5.11 [4.88;5.33] | 0.325 |
| RBP(mg/L) | 41.3 [35.3;43.7] | 40.5 [35.9;42.5] | 0.210 |
| RDW.CV | 12.9 [12.5;13.4] | 13.2 [12.7;13.6] | <0.001 |
| RDW.SD(fL) | 41.4 [40.1;42.2] | 41.1 [40.3;44.4] | 0.124 |
| TBA(μmol/L) | 3.40 [1.90;6.20] | 3.70 [3.00;6.00] | <0.001 |
| TBIL(μmol/L) | 15.9 [12.2;21.5] | 12.0 [10.4;18.0] | <0.001 |
| TP(g/L) | 73.7 [70.7;75.5] | 72.0 [70.5;74.6] | <0.001 |
| UA(μmol/L) | 377 [328;413] | 383 [320;427] | 0.325 |
| Urea(mmol/L) | 5.60 [5.00;6.40] | 5.10 [4.50;5.62] | <0.001 |
| WBC(*10^9^/L) | 5.91 [5.41;7.26] | 6.81 [5.91;7.36] | <0.001 |
| WBC/LYM | 2.90 [2.55;3.25] | 2.70 [2.60;3.10] | 0.011 |
| PLT/LYM | 99.0 [87.1;120] | 95.2 [88.2;118] | 0.053 |

Continuous data conforming to a normal distribution were reported as mean (SD) and those not conforming as median(quartiles). Statistical significance was determined using t-test, Rank-sum test or χ² test, with p-values calculated.Non-High Altitude: an altitude <2500m. High Altitude: an altitude≥2500m. BMI: body mass index. SBP: systolic blood pressure. DBP: diastolic blood pressure. MAP:mean artery pressure.HR: heart rate. OS: oxygen saturation. SCOPA-AUT: the Scale for Outcomes in Parkinson′s Disease for Autonomic Symptoms. A/G: albumin/globulin ratio. ALB:albumin. ALP: alkaline phosphatase. ALT: alanine transaminase. AST:aspartate amino transferase. Baso:basophile granulocyte. Baso.Per: basophile granulocyte percentage. Ca: calcium. CHE: Cholinesterase. Cl:chlorine. CMVD: Coronary Microvascular Disease. CO_2_CP: carbon dioxide binding capacity. Crea:creatinine. CysC: serum cystatin C. DBIL: direct bilirubin. Eo: eosinophils. Eos.Per: eosinophils percentage. FTND:Fagerström Test for Nicotine Dependence.G: globulin. GGT: γ-glutamyl transpeptidase. GLU: glucose. HCT:Hematocrit. HGB: hemoglobin. K: kalium. LCR.Per:Percentage of large platelets. LyM: lymphocytes. Lymph.per: lymphocytes percentage.

MCH:mean corpuscular hemoglobin.MCHC: mean corpuscular hemoglobin concentration. MCV:Mean Corpuscular Volume.Mono:monocytes. Mono.Per: monocytes percentage. MPV:mean platelet volume.Na:Natrium. Neu:neutrophils. Neu.Per: neutrophils percentage. P: phosphorus. PA: Prealbumin. PCT: thrombocytocrit. PDW: platelet distributing width. PLT: platelet. PSQI:The Pittsburgh Sleep Quality Index. RBC:red blood cell. RBP:retinol conjugated protein. RDW.CV: Coefficient of variation of red blood cell distribution width. RDW.SD: Standard deviation of red blood cell distribution width. TBA: total bile acid. TBIL: total bilirubin.TP: total protein. UA: Uric acid. Urea: WBC: white blood cell.WBC/LYM:white blood cell/lymphocytes ratio. PLT/LYM:platelet/lymphocytes ratio.

^*^Participants entered the destination after 1 month of highland acclimatization training at an altitude of 3000m.

^#^ The calculation of physical ability scores was based on Military Common Subject Training Program.


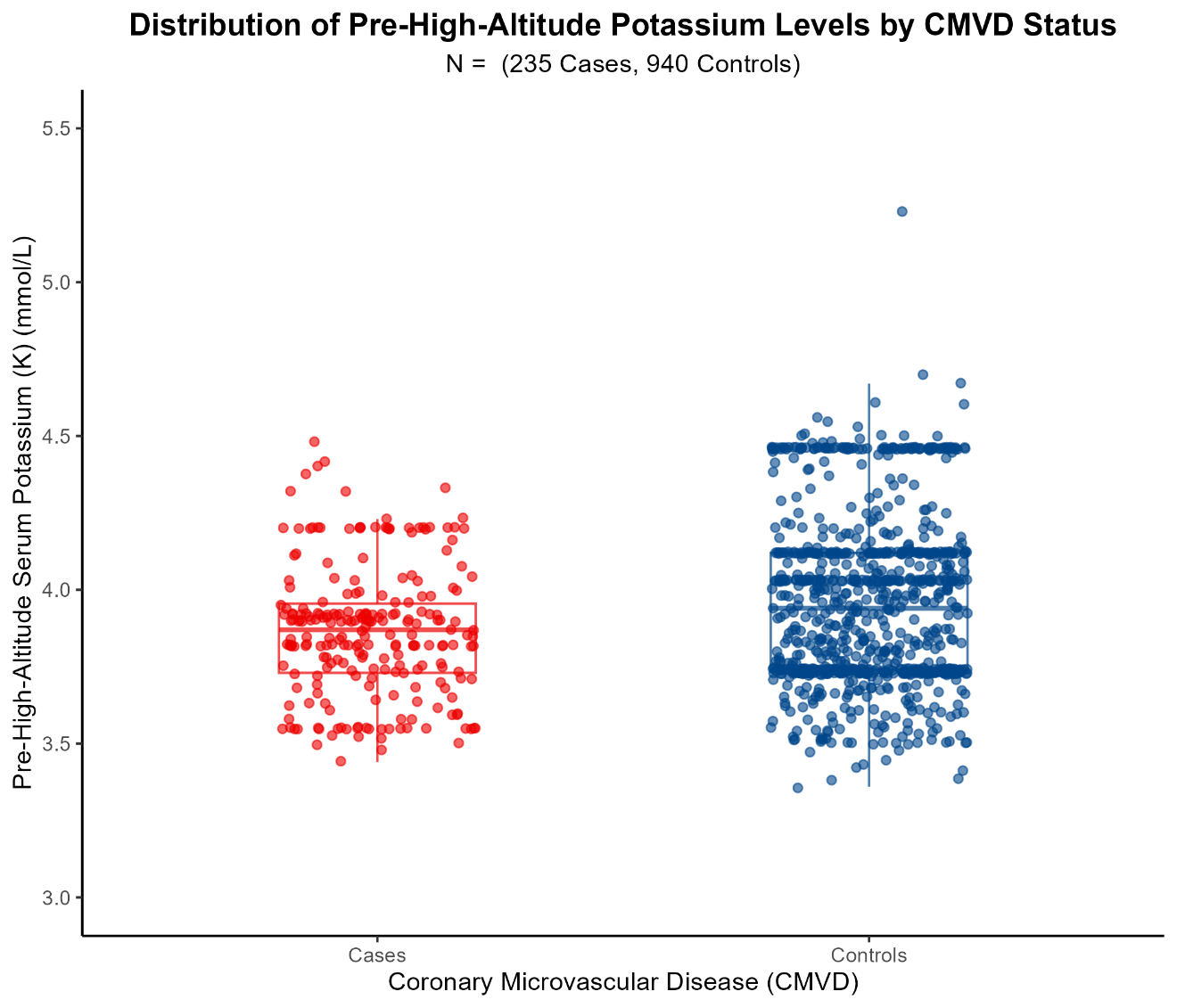


**Figure S1.** **Distribution of pre-high-altitude serum potassium levels in individuals with and without Coronary Microvascular Disease (CMVD).** The figure presents boxplots overlaid with jitter plots, comparing baseline serum potassium concentrations between participants who were subsequently diagnosed with CMVD (Cases, n=235, shown in red) and those who were not (Controls, n=940, shown in blue). The central line in each boxplot represents the median, the box indicates the interquartile range (IQR), and the whiskers extend to 1.5 times the IQR. Each dot represents an individual participant. The visualization demonstrates that the CMVD group had a tendency towards lower baseline potassium levels compared to the control group.
