# Supplementary material for: Inverse association between pre-high-altitude potassium levels and coronary microvascular disease at high altitude: a case-control study
Source: Front Pharmacol. 2025 Aug 21;16:1659041. doi: 10.3389/fphar.2025.1659041 (PMC12408258; doi:10.3389/fphar.2025.1659041)
Supplement: Supplementary file 2 [file DataSheet1.pdf]

International Parkinson and Movement Disorder Society

SCOPA-AUT

Scales for Outcomes in Parkinson's disease - Autonomic Dysfunction

Authored by:
Martine Visser
Johan Marinus
Anne M. Stiggelbout
Jacobus J. van Hilten

Tel +1 (414) 276-2145
Fax +1 (414) 276-3349

555 E. Wells Street, Suite 1100
Milwaukee, WI 53202-3823

www.movementdisorders.org
ratingscales@movementdisorders.org

Copyright © 2019 International Parkinson and Movement Disorder Society. All rights reserved.

MDS Permissions

The Scales for Outcomes in Parkinson's disease - Autonomic Dysfunction (SCOPA-AUT) is owned and licensed by the International Parkinson and Movement Disorder Society (MDS). Permission is required to use the scale and can be obtained by submitting a Permissions Request Form on the MDS website. For licensing inquiries, please e-mail ratingscales@movementdisorders.org.

Unauthorized reproduction, distribution, translation, or sale of any portion of the SCOPA-AUT is strictly prohibited. Changes, modifications, and derivative works of the scale are not permitted without the express authorization of MDS. Including but not limited to the following, the SCOPA-AUT may not be incorporated into clinical trials, training materials, certification programs, software programs, electronic platforms, electronic medical records, databases, or devices except by permission of MDS.

Copyright © 2019 International Parkinson and Movement Disorder Society. All rights reserved.

SCOPA-AUT

By means of this questionnaire, we would like to find out to what extent in the past month you have had problems with various bodily functions, such as difficulty passing urine, or excessive sweating. Answer the questions by placing a cross in the box which best reflects your situation. If you wish to change an answer, fill in the 'wrong' box and place a cross in the correct one. If you have used medication in the past month in relation to one or more of the problems mentioned, then the question refers to how you were while taking this medication. You can note the use of medication on the last page.

---

1. In the past month, have you had difficulty swallowing or have you choked?

☐ never ☐ sometimes ☐ regularly ☐ often

2. In the past month, has saliva dribbled out of your mouth?

☐ never ☐ sometimes ☐ regularly ☐ often

3. In the past month, has food ever become stuck in your throat?

☐ never ☐ sometimes ☐ regularly ☐ often

4. In the past month, did you ever have the feeling during a meal that you were full very quickly?

☐ never ☐ sometimes ☐ regularly ☐ often

5. *Constipation is a blockage of the bowel, a condition in which someone has a bowel movement twice a week or less.*
   In the past month, have you had problems with constipation?

☐ never ☐ sometimes ☐ regularly ☐ often

6. In the past month, did you have to strain hard to pass stools?

☐ never ☐ sometimes ☐ regularly ☐ often

Copyright © 2019 International Parkinson and Movement Disorder Society. All rights reserved.

SCOPA

7. In the past month, have you had involuntary loss of stools?

never	sometimes	regularly	often

Questions 8 to 13 deal with problems with passing urine. If you use a catheter you can indicate this by placing a cross in the box "*use cathether*".

---

8. In the past month, have you had difficulty retaining urine?

never	sometimes	regularly	often	*use catheter*

9. In the past month, have you had involuntary loss of urine?

never	sometimes	regularly	often	*use catheter*

10. In the past month, have you had the feeling that after passing urine your bladder was not completely empty?

never	sometimes	regularly	often	*use catheter*

11. In the past month, has the stream of urine been weak?

never	sometimes	regularly	often	*use catheter*

12. In the past month, have you had to pass urine again within 2 hours of the previous time?

never	sometimes	regularly	often	*use catheter*

13. In the past month, have you had to pass urine <u>at night</u>?

never	sometimes	regularly	often	*use catheter*

Copyright © 2019 International Parkinson and Movement Disorder Society. All rights reserved.

14. In the past month, when standing up have you had the feeling of either becoming lightheaded, or no longer being able to see properly, or no longer being able to think clearly?

☐ never ☐ sometimes ☐ regularly ☐ often

15. In the past month, did you become light-headed after standing for some time?

☐ never ☐ sometimes ☐ regularly ☐ often

16. Have you fainted in the past 6 months?

☐ never ☐ sometimes ☐ regularly ☐ often

17. In the past month, have you ever perspired excessively during the day?

☐ never ☐ sometimes ☐ regularly ☐ often

18. In the past month, have you ever perspired excessively during the night?

☐ never ☐ sometimes ☐ regularly ☐ often

19. In the past month, have your eyes ever been over-sensitive to bright light?

☐ never ☐ sometimes ☐ regularly ☐ often

20. In the past month, how often have you had trouble tolerating cold?

☐ never ☐ sometimes ☐ regularly ☐ often

21. In the past month, how often have you had trouble tolerating heat?

☐ never ☐ sometimes ☐ regularly ☐ often

Copyright © 2019 International Parkinson and Movement Disorder Society. All rights reserved.

SCOPA

The following questions are about sexuality. Although we are aware that sexuality is a highly intimate subject, we would still like you to answer these questions. For the questions on sexual activity, consider every form of sexual contact with a partner or masturbation (self-gratification). An extra response option has been added to these questions. Here you can indicate that the situation described has <u>not been applicable</u> to you in the past month, for example because you have not been sexually active. Questions <u>22 and 23</u> are intended specifically for **men**, <u>24 and 25</u> for **women**.

The following 3 questions are only for men

22. In the past month, have you been impotent (unable to have or maintain an erection)?

☐ never ☐ sometimes ☐ regularly ☐ often ☐ *not applicable*

23. In the past month, how often have you been unable to ejaculate?

☐ never ☐ sometimes ☐ regularly ☐ often ☐ *not applicable*

23a. In the past month, have you taken medication for an erection disorder? (If so, which medication?)

☐ no ☐ yes: ____________________________

Proceed with question 26

The following 2 questions are only for women

24. In the past month, was your vagina too dry during sexual activity?

☐ never ☐ sometimes ☐ regularly ☐ often ☐ *not applicable*

25. In the past month, have you had difficulty reaching an orgasm?

☐ never ☐ sometimes ☐ regularly ☐ often ☐ *not applicable*

Copyright © 2019 International Parkinson and Movement Disorder Society. All rights reserved.

The following questions are for everyone

The questions below are about the use of medication for which you may have or have not needed a doctor's prescription. If you use medication, also give the name of the substance.

26. In the past month, have you used medication for:

a. constipation? ☐ no ☐ yes: ______________________________

b. urinary problems? ☐ no ☐ yes: ______________________________

c. blood pressure? ☐ no ☐ yes: ______________________________

d. other symptoms *(not symptoms related to Parkinson's disease)* ☐ no ☐ yes: ______________________________

____

Use of this questionnaire in studies should be communicated to the International Parkinson and Movement Disorder Society (MDS). No changes may be made to the questionnaire without written permission from MDS. Please use the following reference in publications: Visser M, Marinus J, Stiggelbout AM, Van Hilten JJ. Assessment of autonomic dysfunction in Parkinson's disease: the SCOPA-AUT. Mov Disord 2004;19:1306-12.

To request permission or obtain licensing, please submit a Rating Scale Permission Request Form. For further information, please email Ratingscales@movementdisorders.org.

Copyright © 2019 International Parkinson and Movement Disorder Society. All rights reserved.
